# Association Between Physical Activity and Adherence to Nutritional Recommendations in Individuals with Diabetes: Analysis of Self-Reported Data from the 2020 European Health Survey in Spain

**DOI:** 10.3390/nu17081382

**Published:** 2025-04-19

**Authors:** Carlos Llamas-Saez, Rodrigo Jiménez-García, Luyi Zeng-Zhang, José J. Zamorano-León, Natividad Cuadrado-Corrales, David Carabantes-Alarcón, Andrés Bodas-Pinedo, Ana López-de-Andrés, Ana Jimenez-Sierra, Noemí Serra-Paya

**Affiliations:** 1 Department of Public Health and Maternal & Child Health, Faculty of Medicine, Universidad Complutense de Madrid, Fundación para la Investigación Biomédica del Hospital Clínico San Carlos (IdISSC), 28040 Madrid, Spain; carlolla@ucm.es (C.L.-S.); josejzam@ucm.es (J.J.Z.-L.); mariancu@ucm.es (N.C.-C.); dcaraban@ucm.es (D.C.-A.); abodas@ucm.es (A.B.-P.); 2 Endocrinology and Nutrition Department, Infanta Leonor University Hospital, Universidad Complutense de Madrid, 28031 Madrid, Spain; luyizeng@ucm.es; 3 Department of Public Health and Maternal & Child Health, Faculty of Pharmacy, Universidad Complutense de Madrid, Fundación para la Investigación Biomédica del Hospital Clínico San Carlos (IdISSC), 28040 Madrid, Spain; anailo04@ucm.es; 4 Faculty of Medicine, Universidad San Pablo Ceu, 28668 Madrid, Spain; a.jimenez100@usp.ceu.es; 5 Grupo de Investigación DAFNiS (Dolor, Actividad Física, Nutrición y Salud), Universitat Central de Catalunya (UVIC-UCC), C/Sant Benito Menni, 18-20, 08830 Sant Boi de Llobregat, Spain; noemi.serra@sjd.edu.es

**Keywords:** diabetes, nutrition, physical activity, gender, adherence, lifestyles

## Abstract

**Background/Objectives:** This study compares dietary and physical activity (PA) habits between Spanish adults with and without diabetes and analyzes the association between PA, sociodemographic variables, and adherence to nutritional recommendations among individuals with diabetes. **Methods:** A cross-sectional case–control study was conducted using data from the 2020 European Health Survey for Spain (EESE). Diabetes and PA levels were self-reported. Each participant with diabetes was matched with a control without diabetes by age, gender, and region of residence. Food intake was assessed using a food frequency questionnaire, and adherence to nutritional guidelines was evaluated based on the recommendations of the Spanish Agency for Food Safety and Nutrition. The PA levels were classified as “sedentary/low” or “moderate/high”. **Results:** A total of 2053 matched pairs were analyzed. The participants with diabetes adhered to significantly more nutritional recommendations than those without diabetes (6.19 vs. 5.30; *p* < 0.001). However, 88.6% of the individuals with diabetes reported sedentary or low PA levels. Among those with diabetes, women showed better adherence to nutritional recommendations, while men reported higher PA levels. Moderate/high PA was associated with greater adherence to nutritional recommendations (OR 1.991; 95% CI: 1.201–3.146). Older age was also positively associated with adherence. **Conclusions:** Although individuals with diabetes demonstrated better adherence to nutritional recommendations than controls, most reported low PA levels. Higher PA levels, female gender, and older age were linked to greater adherence to nutritional recommendations among people with diabetes. However, the use of self-reported data made it impossible to judge whether the participants under- or over-reported their PA levels and diabetes status. Public health strategies should aim to promote both PA and healthy eating habits in this population.

## 1. Introduction

Diabetes is a chronic condition with great impact on the quality of life of millions of people and impose a substantial socioeconomic burden [1]. Diabetes prevalence continues to rise, currently affecting nearly 537 million adults worldwide [1]. In Spain, the prevalence reaches 14.8% in the adult population, affecting approximately 5.1 million adults, with a significant proportion of undiagnosed cases [1]. According to the di@bet.es study, the national cumulative incidence of these condition was 6.4% over 7.5 years of follow-up among adults, increasing with age and being higher in males [2].

Managing diabetes requires a multifactorial approach aimed at achieving glycemic control and reducing cardiovascular risk [3]. Among the most cost-effective strategies are therapeutic adherence, appropriate nutritional guidelines, physical activity (PA), and smoking cessation [4].

PA plays a fundamental role in both prevention and management of diabetes, as it improves insulin sensitivity, reduces HbA1c levels, contributes to weight control, and lowers overall cardiovascular risk [5]. However, the PA levels tend to be lower among adults with diabetes compared to the general population. Although there has been a favorable trend in PA engagement over time, a high prevalence of sedentary behavior persists [6]. The sociodemographic and clinical factors associated with lower PA levels among people with diabetes include gender, age, lower educational level, smoking, chronic conditions such as obesity, mental health disorders, and self-perceived health [6].

Diet is equally crucial in diabetes management [7]. The promotion of the “diabetes plate” method is recommended to ensure a nutritious, balanced diet with moderate calorie intake, often stricter than for the general population [8,9]. A personalized dietary plan, with specific recommendations for carbohydrate intake tailored particularly to the PA levels, is encouraged. When coordinated with medication, this approach—referred to as “medical nutritional therapy”—aims to maintain stable blood glucose levels, prevent hypoglycemia, and reduce long-term complications. Additionally, adherence to dietary guidelines contributes to a reduction in cardiovascular comorbidities by decreasing saturated fat and salt intake, thus preventing dyslipidemia and hypertension [7]. Various dietary patterns have demonstrated benefits in metabolic disease management, notably, the Mediterranean diet (MD), which significantly reduces chronic low-grade inflammation and improves insulin sensitivity [10,11,12].

The MD has been designated as an intangible cultural heritage with particular lifestyle and eating habits typical of the Mediterranean Sea region [12,13,14]. The MD is not just a model of cultural food choices, cooking methods, meal patterns, or lifestyle. Several other factors contribute to the MD, such as the selection and preference of a wide variety of traditional and local foods sustained by the local culinary heritage, the use of indigenous ingredients, and thus the production (plants and animal) and capture (e.g., fish, wild rabbit, fowl) of foods that are locally frequent. In the MD, a moderate consumption of wine is also culturally accepted, preferably during a meal. In Spain and other Mediterranean countries, mealtimes have a social and cultural value that transcends their nutritional and nourishing functions. Spain is a major producer and exporter of typical Mediterranean products [13,14].

The MD dietary pattern is characterized by the preferred use of olive oil; a rich intake of plant-based foods; a preference for white lean meat; and a moderate consumption of fish, whole-grain cereals, and red wine [12,13,14,15]. This is additionally balanced by a reduced intake of sugar-sweetened drinks, red and processed meats, whole-fat dairy products, and industrial desserts [12,13,14,15]. However, adherence to this traditional pattern appears to be declining, especially among younger generations, due to the Westernization of diets and the spread of urban lifestyles [14,16]. This change is characterized by an increased consumption of refined cereals, red and processed meat, and beverages containing high levels of sugars [13,14,15,16].

In Spain, adherence to the MD among people with diabetes has been analyzed by different authors, who observed that only a small proportion of these patients have high adherence to the MD, with most patients reaching only a moderate level [17,18,19,20,21,22]. Factors like older age, female gender, high educational level, and living in southern regions were associated with a higher adherence to the MD [17,18,19,20,21,22].

Quarta et al. [15] compared the adherence patterns to the MD in Spain with those in four Mediterranean Southern European countries (Portugal, Italy, Greece, and Cyprus) and two non-Mediterranean European countries (Bulgaria and the Republic of North Macedonia) using the 14-item MD Adherence Screener scoring system. The sample population from Spain showed the highest adherence among all the participant countries [15]. The study groups participating in the European Prospective Investigation into Cancer and Nutrition showed only small differences in the consumption of vegetables, fish, and meat, with slightly higher consumption by individuals with diabetes compared to those without. Among those with diabetes, the findings were more consistent across different ethnic groups than across different countries but generally showed largely similar patterns [23].

Other studies across Europe also suggest that individuals with diabetes in Mediterranean countries, such as Spain or Italy, tend to report higher adherence to healthy dietary patterns, particularly the MD, than those in Northern European countries [24,25,26,27].

To date, national studies have reported varying degrees of adherence to nutritional recommendations among individuals with diabetes, generally demonstrating greater adherence compared to the general population. However, the results concerning associations with variables such as age, gender, and PA are scarce or have not been consistent [17,18,19,20,21,22].

The objectives of this study, based on data from the 2020 European Health Survey for Spain (EESE), are the following: (i) to describe and compare dietary habits and PA levels among Spanish adults with and without diabetes matched by age and gender; (ii) to analyze the association of PA and sociodemographic variables with adherence to nutritional recommendations in individuals with diabetes.

## 2. Materials and Methods

A cross-sectional, case–control study design was employed, matching individuals with and without diabetes by age, sex, and region of residence and improving study efficiency and precision [28].

The data source was the latest EESE conducted in 2020 [29,30]. This survey used a three-stage sampling method to obtain a nationally representative sample of non-institutionalized individuals aged ≥15 years [29]. The participants were personally interviewed at home. The EESE 2020 was conducted between July 2019 and July 2020. Due to the COVID-19 pandemic, telephone interviews were conducted from March to July 2020 [29].

The study population included all interviewed adults (≥18 years). The participants were classified as having diabetes if they answered affirmatively to the question: “*Have you been diagnosed with diabetes by a physician*?” Participants answering “no” were classified as not having this condition.

For each participant with diabetes(case), a control without diabetes, matched by identical age, gender, and region of residence, was randomly selected. If more than one control was available, the selection was made randomly.

### 2.1. Study Variables

The “PA Frequency” variable was created using the EESE question: “*What type of PA do you engage in during your free time?*”, with four possible responses: 1. I do not exercise. My leisure time is primarily sedentary; 2. I engage in occasional physical or sports activities (e.g., walking, cycling, gardening, light gymnastics, recreational activities requiring minimal effort, etc.); 3. I participate in physical activities several times a month (e.g., sports, gymnastics, running, swimming, cycling, team games, etc.); 4. I undergo athletic or physical training multiple times a week. Responses 1 and 2 were categorized as “sedentary or low PA”, and responses 3 and 4 as “moderate or high PA. We joined responses 1 and 2 because, in our opinion, these two options possibly included mostly sedentary individuals. This is so because, in Spanish, “occasional” is a synonym for “sporadic”, “circumstantial”, “fortuitous”, “punctual”. On the other hand, we joined responses 3 and 4 due to the small numbers in these categories among the participants with diabetes (corresponding to 4.7% and 6.7%, respectively) and to the fact that, in our opinion, these adults were clearly not sedentary and performed some regular PA.

The EESE 2020 food frequency questionnaire was utilized [29]. The frequency of consumption of nine food groups was analyzed, including fruits and vegetables; meat; eggs; fish; legumes; pasta, rice, and potatoes; bread and cereals; dairy products; and sugary soft drinks. The *“recommended intakes”* for each food group were those published in the year 2022 by the Agencia Española de Seguridad Alimentaria y Nutrición (Spanish Agency for Food Safety and Nutrition) in the document titled “Informe del Comité Científico de la Agencia Española de Seguridad Alimentaria y Nutrición (AESAN) sobre recomendaciones dietéticas sostenibles y recomendaciones de actividad física para la población Española” (“Report of the Scientific Committee of the Spanish Agency for Food Safety and Nutrition (AESAN) on sustainable dietary recommendations and PA recommendations for the Spanish population”). The details are available elsewhere [31]. Table S1 details the EESE 2020 questions, responses, and categorization criteria used in creating the variables for this study. Additionally, the total number (0–9) of recommendations met was calculated, and the proportion of cases and controls meeting five or more recommendations was analyzed.

The covariates included gender (male/female), age groups (18–49, 50–69, ≥70 years), educational level (no studies/primary, secondary, high education), and living with a partner (see Table S1).

### 2.2. Statistical Analysis

A descriptive analysis was performed using absolute numbers and percentages for the qualitative variables and means with standard deviations for the quantitative variables. Analytical statistics included chi-squared test with Fisher correction, Student’s *t*-test, Mann–Whitney test, ANOVA, or Kruskal–Wallis test, as appropriate. A two-sided *p*-value of <0.05 was considered significant. The Bonferroni–Holm method was applied to adjust the *p* values for multiple testing when required.

Three multivariable logistic regression models (diabetes group, non-diabetes control group, and total population) were constructed, with adherence to more than half of the nutritional recommendations as the dependent variable. The independent variables included gender, age, education level, living with a partner, and diabetes status in the last model. The associations were quantified using the odds ratios (OR) with 95% confidence intervals (95% CI). The analyses were conducted using SPSS v29.

### 2.3. Ethical Considerations

An anonymized EESE dataset is freely accessible via the National Statistics Institute’s website [32]. According to the Spanish legislation, ethics committee approval was not required for this research.

## 3. Results

### 3.1. Description of the Study Population

A total of 2150 participants aged 18 years or older with self-reported diabetes were identified in the EESE 2020. After excluding those with missing data, 2053 subjects with diabetes remained, each one matched with a control without the condition, resulting in a total study population of 4106 individuals (Table 1). Females constituted 50.3% of the sample, and the mean age was 69.60 years (SD 12.89).

### 3.2. Comparison Between Individuals with and Without Diabetes

Table 1 shows the distribution of the participants with and without diabetes, matched by age and gender, according to the study variables. Those with diabetes had significantly lower educational levels and significantly higher sedentary or low PA levels (88.6% vs. 82.6% in the control group; *p* < 0.001).

The adherence to nutritional recommendations was above 75% for all food groups except for fish (45.2%), pasta, rice, and potatoes (6.6%), and legumes (6.6%). The individuals with diabetes showed significantly higher adherence than the control group for the consumption of eggs (92.3% vs. 90.2%) and sugary soft drinks (77.2% vs. 61.5%).

The mean number of nutritional recommendations fulfilled was significantly higher among those with diabetes compared to the control group (6.19 vs. 5.30; *p* < 0.001). More than 90% of the individuals with diabetes met more than half of the total nutritional recommendations, compared to 76.91% in the control group (*p* < 0.001).

### 3.3. Analysis of Adherence to Nutritional Recommendations by Gender

As can be seen in Table 2, males with diabetes exhibited significantly higher adherence to the recommendations than males in the control group for the consumption of eggs (92.6% vs. 89.9%; *p* = 0.028) and sugary soft drinks (77.3% vs. 57.3%; *p* < 0.001).

Among females with diabetes, adherence to sugary soft drink consumption recommendations was significantly higher compared to that of their counterparts in the control group (77.1% vs. 65.3%; *p* < 0.001), though their adherence to the recommended fish consumption was lower (44% vs. 50.4%; *p* = 0.004).

Females with diabetes had higher adherence to the fruit and vegetable, meat, and dairy recommendations, but lower adherence to the recommendations for bread and cereals compared to males with diabetes. The mean number of recommendations met and the proportion of participants meeting more than half of the recommendations were significantly higher among females compared to males with diabetes (Table 2).

### 3.4. Physical Activity Frequency and Adherence to Nutritional Recommendations

Table 3 and Table 4 compare the demographic variables and food consumption by the PA levels across the study groups. Among those with diabetes, individuals with moderate or high PA levels had significantly greater adherence to the dietary recommendations for fruits and vegetables, dairy products, and sugary soft drinks. They also met a significantly higher mean number of recommendations and showed higher adherence rates for more than half of the recommendations.

However, adherence remained low for legumes (9.5%), pasta, rice, and potatoes (6.6%), and fish (45.7%), even among those with higher PA levels.

As can be seen in Table 4, among the matched controls without diabetes, those with sedentary/low PA levels had a significant higher adherence to dietary recommendations than those with moderate/high PA levels only for the consumption of eggs (90.9% vs. 87.3%; *p* = 0.039). Neither the mean number of nutritional recommendations nor the proportion of five or more nutritional recommendations achieved was associated with the level of PA among the participants without diabetes

### 3.5. Multivariable Analysis of Factors Associated with Higher Adherence to Nutritional Recommendations

Table S2 presents a bivariate analysis identifying the variables associated with fulfilling more than half of the nutritional recommendations by the study groups. The multivariable logistic regression results are presented in Table 5.

Older age and female gender were associated with higher adherence to the recommendations across all study groups; females with diabetes had an OR of 1.342 (95% CI: 1.128–1.590) compared to males.

Among those participants with diabetes, moderate to high PA levels were associated with better adherence to nutritional recommendations (OR 1.991; 95% CI: 1.201–3.146). Overall, diabetes was linked to significantly higher adherence to nutritional recommendations (OR 1.692; 95% CI: 1.427–2.006) after multivariable adjustment.

## 4. Discussion

The main findings of our research include the observation of greater adherence to nutritional recommendations among adults with diabetes compared to those without it, across both genders. Conversely, adherence to PA recommendations was lower among individuals with diabetes. Despite females with diabetes being older and having lower educational levels and a higher likelihood of living alone, this group demonstrated greater adherence to nutritional recommendations than their male counterparts. However, males exhibited higher adherence to the recommended PA levels. Among the participants with diabetes, higher PA levels were associated with healthier dietary habits compared with lower PA levels. Age was also positively correlated with healthier dietary consumption in both study groups.

In developed countries, adherence to treatment in people with diabetes averages around 50% [33]. A person is considered adherent if they comply with 80–100% of the prescribed treatments, including medication, dietary recommendations, exercise, and self-management strategies [33,34]. Studies comparing dietary habits between individuals with diabetes and the general population have reported findings consistent with ours [17,18,20,21,22,23,35,36].

An Italian study by Tatoli et al., including 187 adults with diabetes and controls, reported greater dietary differences, with healthier choices for those with diabetes, whose dietary patterns were described as “vegetarian” [35].

These dietary differences have also been studied using healthy diet adherence indicators [37]. Huffman et al. [36] analyzed African American and Haitian American populations with and without diabetes, finding healthier dietary patterns for those with diabetes (N = 250) using the Alternative Healthy Eating Index, based on data from Harvard’s food frequency questionnaire. Additionally, the dietary patterns were less favorable among African Americans compared to Haitian Americans, highlighting the importance of considering ethnicity in health studies [36].

However, a cross-sectional analysis of 111 Irish adults found significantly lower Mediterranean dietary pattern scores among individuals with diabetes compared to controls, measured by the MD Scale and the Alternative MD Scale [38]. In Spain, Vidal-Peracho et al., with a sample of 197 adults with diabetes and controls, reported higher adherence scores for the MD among those with diabetes [17]. Similarly, Alcubierre et al. reported healthier dietary patterns among 238 individuals with diabetes, using the Alternative MD Index and the Alternative Healthy Eating Index [18].

### 4.1. Gender-Specific Analysis

In our gender-specific analysis, females showed consistently higher dietary adherence than males in all population subgroups. Identifying improvement targets for females is particularly important, given that diabetes impacts males and females differently, disproportionately affecting female health across their lifespan [39,40,41].

A cross-sectional study of 2573 Italian adults with diabetes enrolled in the TOSCA.IT study identified gender differences in dietary choices, adherence to nutritional guidelines, and lipid profiles [24]. Further research by this group showed significantly higher MD adherence among females, older individuals, and residents of southern regions, though no associations were found with educational level, smoking, or marital status [25]. Zanetti et al., in a study involving 423 individuals with diabetes, reported higher dietary adherence among females, individuals with higher educational levels, and those with a lower socioeconomic status [26].

National studies, including those by Vidal-Peracho et al. and Alcubierre et al., have similarly reported healthier dietary patterns among females with diabetes compared to males [17,18], as well as with increasing age [18]. Cabrera et al., analyzing lifestyle factors in the CDC Canarias cohort, also found lower therapeutic adherence among males [42]. Another Spanish study in residents with diabetes in the region of Castilla León observed very high adherence to the Mediterranean diet and reported higher adherence among females [19].

The greater involvement of females in meal planning and preparation might partly explain these gender differences [43]. Furthermore, gender norms and social expectations may also influence gender differences in dietary behavior. In many cultures, women are socialized to prioritize caregiving roles and health-related responsibilities including food preparation [43,44,45]. Women demonstrate more trust in healthy nutrition and a greater engagement in controlling body weight and select healthier food than men do [43,44,45]. The so-called “healthier” nutrition of women is also accompanied by greater nutritional knowledge and competence [43,44,45]. Education plays an important role in healthy nutrition; however, gender differences exist, and even when females have a lower formal education level, nutritional knowledge and engagement in preventive health behaviors remain higher for females than for males [45,46]. Also, females have a higher social pressure to reduce eating-related pleasure and to control body weight [43,44,45]. Wong et al. found that males with diabetes were more likely to eat the same meals as the rest of the family, while female patients were more likely to adjust their meals (i.e., reduce portions) or to prepare a separate meal for themselves to meet their nutritional requirements [43]. These psychosocial and cultural dimensions likely interact to promote healthier eating patterns among women with diabetes than among men with diabetes.

### 4.2. Age and Adherence to Nutritional Recommendations

We found that better adherence to nutritional recommendations was associated with older age among the participants with diabetes. The Western dietary patterns have been shifting away from agricultural products toward unhealthy fats, red and processed meats, and refined sugars [13,14,15,16]. The 2030 agenda promotes supporting local agriculture in developed countries, facilitating access to fresh, healthy, and sustainable food products [47]. Older adults in Mediterranean regions maintain traditional dietary habits more effectively than younger individuals [22,48], who face more challenging working conditions affecting healthy food consumption.

### 4.3. Physical Activity and Adherence to Nutritional Recommendations

Our study also revealed significantly lower PA levels among individuals with diabetes compared to those without, consistent with the available evidence [6]. Other national studies have similarly reported discouragingly low PA prevalence, consistently below the recommended levels [6]. These findings might even underestimate the true situation, as individuals with diabetes tend to overestimate their PA levels [49]. A systematic review reported PA adherence among people with diabetes ranging from 32% to 100%, with a median of 58% [50].

Female gender, older age, lower educational level, comorbidities, and smoking have been associated with lower PA levels in those with diabetes and the general population [6].

We observed higher adherence to nutritional recommendations among physically active individuals with diabetes than among patients with low PA levels. Although this subgroup might better control carbohydrate intake compared to more sedentary individuals, adherence to consumption recommendations for pasta, rice, potatoes, and bread remained low, despite a slightly higher adherence to nutritional recommendations for legumes. Even among the most active individuals, adherence was below 10% for pasta, rice, potatoes, and legumes.

Psychosocial factors may also play a key role in explaining why more physically active individuals with diabetes showed higher adherence to nutritional recommendations [51,52,53]. PA influences physiological processes such as appetite control and psychological aspects such as self-efficacy and body image, leading to greater self-determined motivation and, consequently, improvements in dietary self-regulation [51,52] Additionally, regular PA is known to enhance mood and cognitive function, potentially improving decision-making and reducing emotional eating [53]. Moreover, higher health consciousness and intrinsic motivation among physically active individuals may promote a broader commitment to healthy living, including better dietary choices [54].

Males with diabetes exhibited higher adherence to PA recommendations compared to females, which aligns with the existing literature indicating that males consistently engage in more moderate-to-vigorous PA throughout life [55]. Barriers to PA among females include lower social support and motivation and feelings of embarrassment, whereas males predominantly cite lack of time as a barrier [56].

### 4.4. Public Health Implications

Innovative strategies such as digitally delivered interventions are increasingly used to reduce sedentary behavior and promote PA in adults with diabetes [57,58]. A recent meta-analysis of randomized controlled trials concluded that these interventions increased PA and reduced sedentarism in adults with type 2 diabetes [57]. However, other meta-analyses showed that digital health interventions have not yet demonstrated substantial benefits over traditional care for chronic disease management, including diabetes, in European primary care [58].

In Spain, a 12-week, parallel, single-blind randomized controlled trial was conducted with 123 participants recently diagnosed with T2D to assess whether the Greenhabit (mobile health [mHealth]) behavioral treatment enhances T2D outcomes compared with standard care. The study found that PA significantly increased after the intervention [59]. However, also in our country, a two-arm randomized clinical trial involving primary care patients with T2DM to assess the impact of DiabeText, a new theory-based, patient-centered, mobile health intervention integrated with electronic health records to send tailored short text messages to support T2DM self-management, found that over a 12-month period, no statistically significant improvements were observed in adherence to the Mediterranean diet or in PA [60].

These digital approaches hold promises for overcoming traditional barriers to PA, and future public health strategies should incorporate these tools alongside tailored education to optimize outcomes in diabetes care. However, more research is needed to enhance the effectiveness of digital health interventions, address the current methodological limitations, and explore tailored approaches for both specific patient populations and multimorbid populations [57,58,59,60].

Our results reinforce the value of combining nutritional counseling and PA promotion within an integrated framework for diabetes management [61,62]. Individuals who reported moderate-to-high PA levels also exhibited better adherence to dietary recommendations, suggesting a synergistic relationship that could be leveraged in intervention design. Integrated lifestyle programs that simultaneously target diet and PA—particularly when adapted to cultural and sociodemographic contexts—have shown to be able to reduce diabetes incidence and to improve glycemic control, body composition, and cardiovascular outcomes among diabetes patients [61]. Economic evidence also indicates that these programs are cost-effective [62,63,64].

According to the current recommendations, interventions should include multiple sessions related to diet and PA, delivered in person or by other digital methods. These sessions must be provided within community-based programs, for at least three months, providing personalized lifestyle coaching, counseling, and extended support [61,62].

Our findings highlight the need for scalable, gender-sensitive, and age-appropriate programs that promote both dietary quality and PA in parallel [62,63,64].

### 4.5. Study Strength and Limitations

The strength of our study lies in the use of a large, structured health survey, allowing the analysis of nationally representative samples. This provides valuable epidemiological data on health trends, lifestyle habits, and healthcare utilization, critical for health planning and evaluating public health programs.

However, our study has limitations worth noting. The cross-sectional design precludes causal inference. PA classification and dietary intake relied solely on self-reported frequencies, and therefore misclassification bias, such as recall and social desirability bias, must be considered [65]. The report of PA levels and dietary intake by diabetes patients could be affected by the fact that these patients are probably more strongly counseled by health professionals to engage in PA and a healthy diet than their healthy counterparts. Therefore, the lack of information about the advice that individuals received limits the internal validity of our findings. Furthermore, the lack of objective validation (e.g., accelerometer data) of PA is important and must be considered when interpreting the strength of the associations.

Regarding the validity of self-reported diabetes, a national study demonstrated specificity >95% and sensitivity >70% for self-reported diabetes using medical records as a gold standard [66]. Studies conducted in Spain and other countries agree on the validity of self-reported diabetes for epidemiological investigation [67,68,69]. However, we lacked specific information on diabetes such as type, complications, duration, and treatment. In Spain, the prevalence of type 1 diabetes is estimated to be around two cases per 1000 inhabitants, representing under 4% of all persons with diabetes in our country. Therefore, most adults with diabetes participating in our investigation would have had type 2 diabetes [70,71]. The absence of clinical data such as diabetes type or HbA1c levels limited the ability to stratify patients with disease severity. Future studies should incorporate these clinical endpoints.

We did not analyze the socioeconomic status because, in our country, the correlation of education level with socioeconomic status is high; so, we decided to include the educational level, as this variable is possibly more strongly associated with either PA or dietary habits. Additionally, the EESE does not include urban vs. rural residence data, which may influence dietary and PA patterns [72].

## 5. Conclusions

Spanish adults with diabetes exhibited greater dietary adherence than those without diabetes, though significant dietary gaps remained (e.g., fish, legumes). The PA levels remained critically insufficient among individuals with diabetes. Higher PA levels, female gender, and older age were associated with better adherence to nutritional recommendations. These findings can inform public health strategies promoting healthy lifestyles, emphasizing gender and age differences.

## Figures and Tables

**Table 1:** Distribution of participants with diabetes and matched controls without diabetes according to socio-demographic variables, physical activity level, and adherence to recommended consumption of food groups.

Variables	Categories	No Diabetes		Diabetes		*p*
		N	%	N	%	
Gender	Male	1020	49.7	1020	49.7	NA
	Female	1033	50.3	1033	50.3	
Age groups	18 to 49 years	136	6.6	136	6.6	NA
	50 to 69 years	775	37.8	775	37.8	
	70 or older	1138	55.5	1138	55.5	
Age (mean) [SD]		(69.60)	[12.89]	(69.60)	[12.89]	NA
Living with a partner	Yes	1081	53.0	1065	52.1%	0.573
Educational level	No studies/primary	1360	66.2	1549	75.5%	<0.001
	Secondary	291	14.2	239	11.6%	
	High education	402	19.6	265	12.9%	
Physical activity frequency	Sedentary/low	1695	82.6	1819	88.6%	<0.001
	Moderate/high	358	17.4	234	11.4%	
Adherence to recommended consumption of fruits and vegetables		1635	79.7	1657	80.7	0.433
Adherence to recommended consumption of meat		1609	78.6	1605	78.2	0.790
Adherence to recommended consumption of eggs		1849	90.2	1895	92.3	0.017
Adherence to recommended consumption of fish		979	47.8	928	45.2	0.104
Adherence to recommended consumption of pasta, rice, and potatoes		1706	83.3	1700	82.8	0.739
Adherence to recommended consumption of bread and cereals		124	6.1	140	6.8	0.340
Adherence to recommended consumption of legumes		134	6.5	136	6.6	0.950
Adherence to recommended consumption of dairy products		1732	84.5	1747	85.1	0.602
Adherence to recommended consumption of sugary soft drinks		1259	61.5	1584	77.2	<0.001
Number of nutritional recommendations achieved (mean) [SD]		(5.30)	[1.2]	(6.19)	[1.34]	<0.001
Five or more nutritional recommendations achieved		1569	76.9	1852	90.6	<0.001

NA: not applicable; SD: standard deviation. *p* values for differences between participants with diabetes and matched controls without diabetes.

**Table 2:** Distribution of participants with diabetes and matched controls without diabetes by socio-demographic variables, physical activity levels, and adherence to recommended consumption of food groups according to gender.

		Male					Female				
Variables	Categories	No Diabetes		Diabetes		*p*	No Diabetes		Diabetes		*p*
		N	%	N	%	%	N	%	N	%	
Age groups *	18 to 49 years	74	7.3	74	7.3	NA	62	6.0	62	6.0	NA
	50 to 69 years	447	44.0	447	44.0		328	31.8	328	31.8	
	70 or older	496	48.8	496	48.8		642	62.2	642	62.2	
Age (mean) [SD] *		(67.84)	[12.43]	(67.84)	[12.43]	NA	(71.35)	[13.10]	(71.35)	[13.10]	NA
Living with a partner *	Yes	641	63.3	643	63.2	0.981	440	42.8	422	41.1	0.410
Educational level *	No studies/primary	622	61.0	714	70.0	<0.001	738	71.4	835	80.8	<0.001
	Secondary	157	15.4	145	14.2		134	13.0	94	9.1	
	High education	241	23.6	161	15.8		161	15.6	104	10.1	
Physical activity frequency *	Sedentary/low	822	80.6	885	86.8	<0.001	873	84.5	934	90.4	<0.001
	Moderate/high	198	19.4	135	13.2		160	15.5	99	9.6	
Adherence to recommended consumption of fruits and vegetables *		771	75.7	804	78.8	0.091	864	83.7	853	82.6	0.518
Adherence to recommended consumption of meat		774	76.2	778	76.3	0. 958	835	81.0	827	80.1	0.617
Adherence to recommended consumption of eggs		915	89.9	944	92.6	0.028	934	90.6	951	92.1	0.242
Adherence to recommended consumption of fish		459	45.1	473	46.4	0.564	520	50.4	455	44.0	0.004
Adherence to recommended consumption of pasta, rice, and potatoes		86	8.5	69	6.8	0.156	48	4.7	67	6.5	0.084
Adherence to recommended consumption of bread and cereals *		875	86.0	874	85.7	0.849	831	80.5	826	80.0	0.825
Adherence to recommended consumption of legumes		60	5.9	69	6.8	0.467	64	6.2	71	6.9	0.593
Adherence to recommended consumption of dairy products *		838	82.4	847	83.1	0.682	894	86.6	900	87.1	0.745
Adherence to recommended consumption of sugary soft drinks		585	57.6	788	77.3	<0.001	674	65.3	796	77.1	<0.001
Number of nutritional recommendations achieved (mean) [SD] *		5.29	1.33	6.16	1.36	<0.001	5.53	1.22	6.44	1.33	<0.001
Five or more nutritional recommendations achieved *		765	73.4	896	87.8	<0.001	804	83.2	956	92.4	<0.001

NA: not applicable; SD: standard deviation. *p* values for differences between participants with diabetes and matched controls without diabetes. * Means significant differences (*p* < 0.05) between males and females with diabetes.

**Table 3:** Distribution of participants with diabetes by socio-demographic variables and adherence to recommended consumption of food groups according to physical activity frequency.

		Diabetes				
Variable	Categories	Sedentary/Low		Moderate/High		*p*
		N	%	N	%	
Gender	Male	885	48.6	135	57.5	0.011
	Female	934	51.4	99	42.5	
Age groups	18 to 49 years	104	5.7	32	13.7	<0.001
	50 to 69 years	667	36.7	108	46.4	
	70 or older	1045	57.5	93	39.9	
Age (Mean) [SD]		70.46	12.42	63.84	13.22	<0.001
Living with a partner	No	882	48.6	98	41.8	0.057
	Yes	930	51.4	135	58.2	
Educational level	No studies/primary	1414	77.7%	135	57.5%	<0.001
	Secondary	198	10.9%	41	17.6%	
	High education	207	11.4%	58	24.9%	
Adherence to recommended consumption of fruits and vegetables		1526	79.9	210	86.4	0.016
Adherence to recommended consumption of meat		1507	78.9	186	76.5	0.406
Adherence to recommended consumption of eggs		1764	92.4	221	90.9	0.446
Adherence to recommended consumption of fish		861	45.1	111	45.7	0.891
Adherence to recommended consumption of pasta, rice, and potatoes		126	6.6	16	6.6	1.000
Adherence to recommended consumption of bread and cereals		1572	82.3	205	84.4	0.473
Adherence to recommended consumption of legumes		124	6.5	23	9.5	0.103
Adherence to recommended consumption of dairy products		1614	84.5	219	90.5	0.012
Adherence to recommended consumption of sugary soft drinks		1459	76.4	205	84.4	0.004
Number of nutritional recommendations achieved (mean) [SD]		5.85	1.35	6.48	1.22	<0.001
Five or more nutritional recommendations achieved		1573	86.48	227	97.01	<0.001

SD: standard deviation. *p* values for differences between participants with sedentary/low and participants with moderate/high physical activity levels.

**Table 4:** Distribution of matched controls without diabetes by socio-demographic variables and adherence to recommended consumption of food groups according to physical activity frequency.

		No Diabetes				
Variable	Categories	Sedentary/Low		Moderate/High		*p*
		N	%	N	%	
Gender	Male	822	48.5	198	55.2	0.019
	Female	873	51.5	160	44.8	
Age groups	18 to 49 years	92	5.4	44	12.4	<0.001
	50 to 69 years	609	36.0	166	46.8	
	70 or older	993	58.6	145	40.8	
Age (Mean) [SD]		70.75	12.58	64.73	11.99	<0.001
Living with a partner	No	82	47.6	157	43.6	0.226
	Yes	882	52.4	199	56.4	
Educational level	No studies/primary	1197	70.6	163	45.1	<0.001
	Secondary	219	12.9	72	20.3	
	High education	279	16.5	123	34.6	
Adherence to recommended consumption of fruits and vegetables		1338	79.0	297	83.7	0.047
Adherence to recommended consumption of meat		1337	79.2	272	76.1	0.188
Adherence to recommended consumption of eggs		1537	90.9	312	87.3	0.039
Adherence to recommended consumption of fish		806	47.7	173	48.5	0.795
Adherence to recommended consumption of pasta, rice, and potatoes		109	6.4	25	7.0	0.682
Adherence to recommended consumption of bread and cereals		1398	82.7	308	85.9	0.136
Adherence to recommended consumption of legumes		105	6.2	19	5.4	0.530
Adherence to recommended consumption of dairy products		1432	84.7	300	83.9	0.729
Adherence to recommended consumption of sugary soft drinks		1026	60.7	233	65.4	0.105
Number of nutritional recommendations achieved (mean) [SD]		5.19	1.279	5.45	1.33	0.424
Five or more nutritional recommendations achieved		1312	77.40	267	74.58	0.675

SD: standard deviation. *p* values for differences between participants with sedentary/low and participants with moderate/high physical activity levels.

**Table 5:** Multivariable analysis with logistic regression to identify variables associated with having more than half of the nutritional recommendations met according to the diabetes status.

Variables	Categories	No Diabetes			Diabetes			All Participants		
		OR	CI95%		OR	CI95%		OR	CI95%	
			Inf	Sup		Inf	Sup		Inf	Sup
Gender	Male	Reference	-	-	Reference	-	-	Reference	-	-
	Female	1.540	1.227	1.933	1.342	1.128	1.590	1.425	1.256	1.657
Age groups	18 to 49 years	Reference	-	-	Reference	-	-	Reference	Reference	Reference
	50 to 69 years	2.356	1.595	3.479	1.635	1.061	2.569	1.911	1.287	2.606
	70 or older	3.259	2.101	5.902	1.852	1.468	6.013	2.480	1.627	3.833
Physical activity frequency	Sedentary/low	NS	NS	NS	Reference	-	-	Reference	-	-
	Moderate/high	NS	NS	NS	1.991	1.201	3.146	1.435	1.118	1.842
Diabetes	No	NA	NA	NA	NA	NA	NA	Reference	-	-
	Yes	NA	NA	NA	NA	NA	NA	1.692	1.427	2.006

CI: confidence interval. Inf; inferior. Sup; superior. OR: odds ratio. NS: not significant. NA: not applicable (variable not included in the model).

## Data Availability

The anonymized EESE datasets are freely accessible and can be downloaded by anyone from the Ministry of Health’s website https://www.sanidad.gob.es/estadEstudios/estadisticas/EncuestaEuropea/home.htm (accessed on 24 November 2024). All other relevant data are included in the paper.

## Supplementary Materials

The following supporting information can be downloaded at: https://www.mdpi.com/article/10.3390/nu17081382/s1, Table S1: Definition of variables according to the questions included in the European Health Interview Surveys in Spain conducted in the year 2020; Table S2: Distribution of participants with diabetes and matched controls without diabetes that achieved an adherence to five or more nutritional recommendations according to socio-demographic variables and physical activity level.

## Abbreviations

The following abbreviations are used in this manuscript:

EESE	European Health Survey for Spain
MD	Mediterranean diet
PA	Physical activity
