# Supplementary material for: Association Between Physical Activity and Adherence to Nutritional Recommendations in Individuals with Diabetes: Analysis of Self-Reported Data from the 2020 European Health Survey in Spain
Source: Nutrients. 2025 Apr 19;17(8):1382. doi: 10.3390/nu17081382 (PMC12029994; doi:10.3390/nu17081382)
Supplement: Supplementary file 1 [file nutrients-17-01382-s001.zip › nutrients-3573110-supplementary.pdf]

Table S1. Definition of variables according to the questions included in the European Health Interview Surveys in Spain conducted in year 2020.

**Questions**	**Description and answer**	**Variables name**	**Categories**
Have you been diagnosed with diabetes by a physician?	1.Yes<br>2.No	Diabetes	1. Case<br>2. Control
What type of physical activity do you engage in during your free time?	1. I do not exercise. My leisure time is primarily sedentary.<br>2. I engage in occasional physical or sports activities (e.g., walking, cycling, gardening, light gymnastics, recreational activities requiring minimal effort, etc.).<br>3. I participate in physical activities several times a month (e.g., sports, gymnastics, running, swimming, cycling, team games, etc.).<br>4. I undergo athletic or physical training multiple times a week.	Physical activity frequency	1 and 2. Sedentary/low"<br>3 and 4. "Moderate/high"
Which is your gender?	1. Male<br>2. Female	Gender	1. Male<br>2. Female
How old are you?	Age in years	Age groups	1. 18-49<br>2. 50-69<br>3. ≥70
What level of education have you completed?	1. Does not know how to read or write<br>2. Incomplete primary education<br>3. Complete primary education<br>4. First stage of Secondary Education, with or without a qualification<br>5. Elementary Spanish Upper Secondary Education<br>6. Upper secondary education<br>7. Intermediate vocational training or equivalent<br>8. Advanced vocational training or equivalent<br>9. University studies or equivalent<br>10. Over university (master, PhD….)	Educational level	1. No studies/Primary: Options 1 to 3<br>2. Secondary: Options 4 to 8<br>3. High education: Options 9 and 10
What is your marital status?	1. Single<br>2. Married<br>3. Widower<br>4. Separated<br>5. Divorced	Living with a partner	1. Yes: Option 2<br>2. Nor: options 1, 3, 4 and 5

Table S1. Definition of variables according to the questions included in the European Health Interview Surveys in Spain conducted in years 2014 and 2020. (Continued)

Questions	Description and answer	Variables	Categories
How often do you eat fruit and vegetables?	1. Daily or more than once a day<br>2. 4-6 days per week<br>3. 3 days per week<br>4. 1 or 2 days per week<br>5. Less than once a month<br>6. Never	Adherence to recommended consumption of fruit and vegetables	1. Yes: Options 1<br>2. No: Options 2 to 6
How often do you eat meat.	1. Daily or more than once a day<br>2. 4-6 days per week<br>3. 3 days per week<br>4. 1 or 2 days per week<br>5. Less than once a month<br>6. Never	Adherence to recommended consumption of meat	1. Yes: Options 3 to 6<br>2. No: Options 1 or 2
How often do you eat eggs?	1. Daily or more than once a day<br>2. 4-6 days per week<br>3. 3 days per week<br>4. 1 or 2 days per week<br>5. Less than once a month<br>6. Never	Adherence to recommended consumption of eggs	1. Yes: Options 3 to 6<br>2. No: Options 1 or 2
How often do you eat fish?	1. Daily or more than once a day<br>2. 4-6 days per week<br>3. 3 days per week<br>4. 1 or 2 days per week<br>5. Less than once a month<br>6. Never	Adherence to recommended consumption of fish	1. Yes: Options 1 to 3<br>2. No: Options 4 to 6
How often do you eat legumes?	1. Daily or more than once a day<br>2. 4-6 days per week<br>3. 3 days per week<br>4. 1 or 2 days per week<br>5. Less than once a month<br>6. Never	Adherence to recommended consumption of legumes	1. Yes: Options 1 or 2<br>2. No: Options 3 to 6
How often do you eat pasta, rice, and potatoes?	1. Daily or more than once a day<br>2. 4-6 days per week<br>3. 3 days per week<br>4. 1 or 2 days per week	Adherence to recommended consumption of pasta, rice, and potatoes	1. Yes: Option 1<br>2. No: Options 2 to 6

	5. Less than once a month<br>6. Never		
How often do you eat bread and cereals?	1. Daily or more than once a day<br>2. 4-6 days per week<br>3. 3 days per week<br>4. 1 or 2 days per week<br>5. Less than once a month<br>6. Never	Adherence to recommended consumption of bread and cereals	1. Yes: Option 1<br>2. No: Options 2 to 6
How often do you eat dairy products?	1. Daily or more than once a day<br>2. 4-6 days per week<br>3. 3 days per week<br>4. 1 or 2 days per week<br>5. Less than once a month<br>6. Never	Adherence to recommended consumption of dairy products	1. Yes: Option 1<br>2. No: Options 2 to 6
How often do you drink soft sugary drinks?	1. Daily or more than once a day<br>2. 4-6 days per week<br>3. 3 days per week<br>4. 1 or 2 days per week<br>5. Less than once a month<br>6. Never	Adherence to recommended consumption of sugary soft drinks	1. Yes: Option 6<br>2. No: Option 1 to 5
Number of nutritional recommendations achieved	From 0 to 9	Five or more nutritional recommendations achieved	1. Yes; five or more.<br>2. No: four or less

Table S2. Distribution of participants with diabetes and matched controls without diabetes that achieved an adherence to five or more nutritional recommendations according to socio-demographic variables and physical activity.

VARIABLES	CATHEGORIES	NO DIABETES		DIABETES		ALL PARTICIPANTS	
		Five or more nutritional recommendations achieved		Five or more nutritional recommendations achieved		Five or more nutritional recommendations achieved	
		%	P	%	p	N	p
Gender	Male	73.4	<0.001	87.8	<0.001	81.3	<0.001
	Female	83.2		92.4		87.2	
Age groups	18 to 49 years	56.6	<0.001	73.9	<0.001	65.3	<0.001
	50 to 69 years	73.6		85.5		79.6	
	70 or more years	86.0		93.2		89.6	
Living with a partner	No	76.7	0.864	90.8	0.783	83.8	0.794
	Yes	76.8		90.2		83.5	
Educational level	No studies/Primary	79.5	0.155	91.7	0.051	85.6	0.052
	Secondary	74.8		86.7		80.8	
	High education	77.0		86.9		81.9	
Physical activity frequency	Sedentary/low	77.2	0.708	85.4	0.014	81.3	0.127
	Moderate/high	76.6		93.3		84.9	
Diabetes	No	NA	NA	NA	NA	76.91	<0.001
	Yes	NA		NA		90.61	

NA Not available
